# Lithium Intoxication as a cause of reversible dementia mimicking FDG PET features of Alzheimer’s disease

**DOI:** 10.1590/1980-5764-DN-2021-0105

**Published:** 2022-04-29

**Authors:** Alexandre Motta Mecê, Vitor Corsaletti Abreu, Gustavo Manginelli Lamas, Rafaella do Rosário Tacla, Thais Benício Minekawa, Celso Dario Ramos, Marcio Luiz Figueiredo Balthazar

**Affiliations:** 1 Universidade Estadual de Campinas, Faculdade de Ciências Médicas, Departamento de Neurologia, São Paulo SP, Brazil.; 2 Universidade Estadual de Campinas, Faculdade de Ciências Médicas, Departamento de Radiologia, Divisão de Medicina Nuclear, São Paulo SP, Brazil.

**Keywords:** Lithium, Positron Emission Tomography Computed Tomography, Bipolar Disorder, Lítio, Tomografia por Emissão de Pósitrons combinada à Tomografia Computadorizada, Transtorno Bipolar

## Abstract

Rapidly progressive dementia (RPD) is a rare neurological disorder. Drug toxicity is among the differential diagnoses, including the use of lithium, in which an overdosage might cause cognitive dysfunction. Clinical suspicion, laboratory confirmation, and drug interruption are key points in the management of lithium intoxication. We described a 66-year-old female patient under treatment with lithium who developed an RPD associated with parkinsonian symptoms. ^18^F-fluorodeoxyglucose positron emission tomography/computed tomography (^18^F-FDG PET/CT) showed an “Alzheimer-like” pattern, while cerebrospinal fluid biomarkers for the disease were negative. There was a significant clinical and radiological improvement after lithium interruption. Lithium intoxication is a potentially reversible cause of RPD, as demonstrated in this case report. Drug discontinuation should be considered even in patients with normal levels of this metal, if cognitive impairment is detected. ^18^F-FDG PET/CT images may show an “Alzheimer-like” image pattern in acute intoxication and are useful for monitoring these patients.

## RESUMO.

Demência rapidamente progressiva é uma condição rara, cujos diagnósticos diferenciais incluem intoxicação por drogas como lítio, podendo causar importante disfunção cognitiva. A suspeita clínica, a confirmação laboratorial e a interrupção do uso medicamentoso são elementos fundamentais em seu diagnóstico e manejo. Trata-se de paciente feminina, de 66 anos de idade, que apresentou quadro demencial após intoxicação por lítio. Tal quadro foi acompanhado de sintomas parkinsonianos, além de Tomografia por Emissão de Pósitrons com 18F-Fluodeoxiglicose (^18^F-FDG PET/CT) compatível com padrão “Alzheimer-like”. Houve melhora objetiva de parâmetros clínicos e imaginológicos após a interrupção do uso medicamentoso. A intoxicação por lítio é uma causa potencialmente reversível de demência rapidamente progressiva. A descontinuação da droga deve ser considerada também em pacientes com níveis normais do metal no sangue se alterações cognitivas forem encontradas. Imagens ^18^F-FDG PET/CT podem demonstrar achados sugestivos de doença de Alzheimer na intoxicação aguda e parecem ser um método útil no seguimento.

## INTRODUCTION

Rapidly progressive dementia (RPD) is a neurological condition characterized by a decline in more than one cognitive domain with functional impairment in less than 1–2 years. Specific diagnosis is crucial, since 20–30% of the cases can be related to potentially reversible disorders^1–3^. There are several possible etiologies for RPD, including iatrogenic causes, such as medication toxicity.

Lithium is a widely used drug for preventing manic and depressive recurrences in bipolar disorder (BD). Although lithium is effective in the treatment of this disease, it has a narrow therapeutic ratio, and overdoses are potentially neurotoxic. Despite its potential effect on the prevention of cognitive dysfunction and dementia, high serum lithium levels are associated with the development of RPD^3–5^, and it should be considered among the iatrogenic causes of this condition.

Serum lithium dosage and clinical improvement after treatment interruption are necessary for the diagnosis, and the interruption of the medication can lead to the recovery of dementia symptoms.

We reported a case of lithium intoxication leading to RPD with ^18^F-fluorodeoxyglucose positron emission tomography/computed tomography (^18^F-FDG PET/CT) features of Alzheimer’s disease (AD), which was discarded due to normal cerebrospinal fluid biomarkers. There was a significant improvement in clinical and imaging aspects after lithium’s discontinuation.

## CASE REPORT

A 66-year-old female patient developed an RPD syndrome associated with asymmetric parkinsonian symptoms 6 months before hospitalization. She progressively developed asymmetric resting tremors (initially on the superior right limb), psychomotor slowing, inattention, language problems, echolalia, shuffling gait, and postural instability with frequent falls. After a few weeks, the patient presented with executive and visuospatial dysfunctions, as well as anterograde amnesia and disorientation. An acute episode of diarrhea and dehydration worsened these clinical manifestations. Personal background included hypertension, hypothyroidism, smoking, and chronic pulmonary obstructive disease. The patient was also under psychiatric follow-up at another service due to bipolar affective disorder. Lithium carbonate, prescribed as 600 mg/day, was among the drugs taken for at least 1 year, although we are not sure whether the patient had taken the prescribed dose. Other medications included quetiapine, hydrochlorothiazide, irbesartan, venlafaxine, esomeprazole, amlodipine, clonazepam, tiotropium, and formoterol.

Cognitive and functional examination revealed the following results: Mini-Mental Status Examination=7, Montreal Cognitive Assessment (MoCA)=5, and Pfeffer’s Functional Activities Questionnaire=28. Mixed transcortical aphasia, apraxia, and mild inattention were also present, while resting tremor, postural instability, hypertonia, and bradykinesia were associated with parkinsonian clinical features.

**Figure 1:**
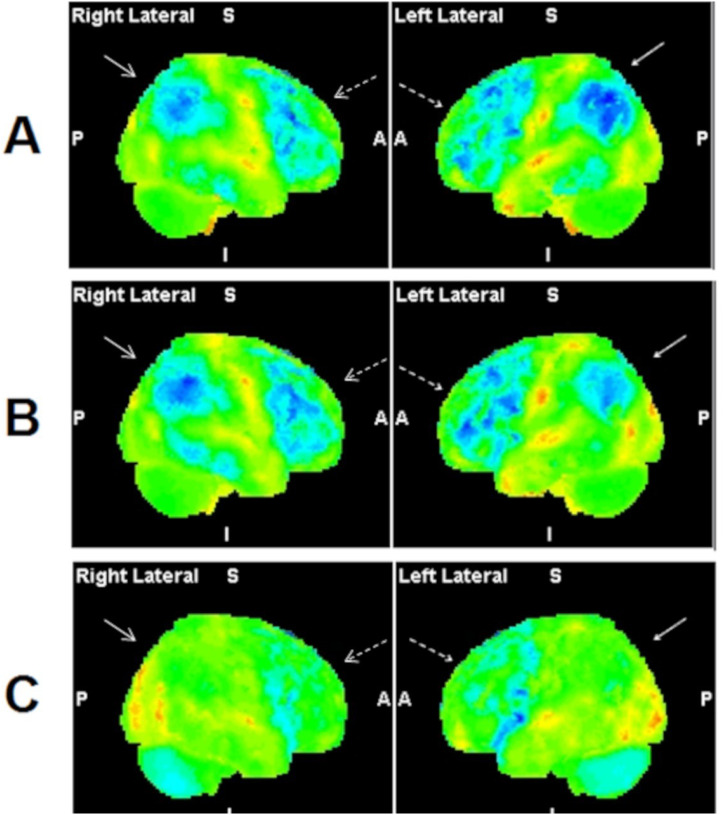
Quantitative analysis of the ^18^F-FDG PET/CT images (Scenium® software, Siemens CTI Molecular Imaging, Knoxville, TN, USA). The software conducts a voxel-by-voxel statistical comparison of patient’s PET/CT with a control group. The quantitative analysis delineates areas of significant hypometabolism (>2 SDs from the mean) and depicts them in color tridimensional images. Regions with normal and reduced glucose metabolism are displayed in green and blue, respectively. (A) Right and left lateral projections of quantitative images obtained during acute lithium intoxication show marked bilateral hypometabolism of the parietal lobes (arrows) and moderate hypometabolism of frontal lobes (dashed arrows). (B) Images obtained on the sixth day after lithium withdrawal show a significant improvement of glucose metabolism in the left parietal lobe (arrow), with hypometabolism persisting in the right parietal and frontal lobes (dashed arrows). (C) Two months after lithium discontinuation, complete resolution of glucose metabolism is evident in the parietal lobes (arrows), with only mild hypometabolism of the frontal lobes persisting (dashed arrows).

After the initial ambulatory consultation, the patient was hospitalized aiming for a diagnostic workup. Several screening tests were then performed: simple blood tests and metabolic panel were normal; brain magnetic resonance imaging (MRI) revealed white matter microangiopathy (Fazekas scale=2); ^18^F-FDG PET/CT demonstrated severe bilateral hypometabolism of the parietal lobes, including the precuneus, and moderate hypometabolism of the frontal lobes, the common findings in AD (Figure 1A). Of note, there was no hypometabolism of the posterior cingulate gyrus and temporal lobes, both of which were usually involved in AD. Cerebrospinal fluid tested negative, including normal AD biomarkers — amyloid beta=661 ng/L (normal: 562–1018), phosphorylated Tau=30.84 ng/L (normal: 35.84–66.26), and slightly elevated total Tau protein=398 ng/L (normal: 116–370). High lithium serum levels were detected (2.6 mmol/L; normal: 0.5–1.20 mmol/L). These findings that were associated with cognitive and parkinsonian clinical features suggested lithium intoxication.

Six days after lithium withdrawal, serum levels decreased to 1.48 mmol/L and clinical improvement was observed. ^18^F-FDG PET/CT was repeated, and the images showed marked improvement of glucose metabolism in the left parietal lobe, including the precuneus. However, hypometabolism persisted in right parietal lobe and frontal lobe (Figure 1B). Four days later (a total of 10 days without lithium), the patient showed a significant improvement in attention, memory, and executive functions. There was also an improvement in the MoCA score from 5 to 14 points, as well as almost complete remission of the symptoms of parkinsonism. The patient was discharged from the hospital and was scheduled for outpatient follow-up.

Thirty days after lithium withdrawal, the patient returned to consultation presenting with complete resolution of parkinsonian symptoms. MoCA score was 23/30. Executive and visuospatial function and memory had substantially improved. New PET/CT performed for 2 months after lithium discontinuation showed a complete resolution of the parietal lobes, including the precuneus, with only mild hypometabolism of the frontal lobes persisting (Figure 1C).

## DISCUSSION AND LITERATURE REVIEW

Several studies have evaluated the cumulative lithium effects in the central nervous system. Although BD patients are at increased risk of developing dementia, intake of chronic lithium seems to present a protective effect^4,6^.

However, treatment with lithium may be harmful in some circumstances. The particular pharmacokinetics of the drug can make its therapeutic and toxic levels very close to each other. Intoxication is a relatively common event, especially in patients with dehydration and renal failure. Patients using other medications are at higher risk of intoxication due to drug interaction. Both acute and chronic forms may occur, depending on the dose of lithium and the patient’s risk factors^1,7^. In this case report, an important cognitive dysfunction occurred after at least 12 months of lithium intake, with worsening of cognitive symptoms after an episode of diarrhea and dehydration. Although the patient evolved with complete resolution of parkinsonian symptoms and with improvement in executive/visuospatial functions and memory, the mild cognitive deficit persisted. In fact, glucose metabolism of the frontal lobes remained reduced, despite a complete normalization of the parietal metabolism, as shown by PET/CT images.

Acute lithium intoxication may present with gastrointestinal (e.g., nausea, vomiting, and diarrhea), cardiovascular (e.g., bradycardias and QT interval prolongation), and neurological manifestation (e.g., ataxia, tremors, myoclonus, fasciculations, confusion, and even encephalopathy). Nevertheless, patients who were chronically intoxicated might present the Syndrome of Irreversible Lithium Effectuated Neurotoxicity (SILENT) syndrome. It is associated with high lithium doses and a lack of improvement after drug discontinuation. Symptoms include parkinsonism, dementia, cerebellar, and brainstem dysfunction^1,7^; they may persist over months or years after lithium interruption. This case report, as well as others in the literature, presented an association between RPD and extrapyramidal symptoms. Within 30 days of lithium withdrawal, the patient had substantial clinical improvement, with significant recovery of cognitive functions, differently than the expected in SILENT syndrome^8,9^.

The diagnosis of lithium intoxication is based on clinical features and serum lithium levels. A high dosage strongly suggests intoxication. In contrast, some patients may evolve with RPD syndrome and normal serum lithium levels with clinical improvement after discontinuation of medication. In this clinical case, treatment with lithium dosage was performed on the peak of cognitive manifestations, highly suggesting toxicity. Thus, in the case of patients who take lithium chronically, even with normal lithium levels, the interruption of the medication should be considered if the patient presents cognitive symptoms. Soni^9^ described a patient who had been taking lithium for 25 years and developed cognitive manifestations despite the 18-month normal serum levels, suggesting that high lithium levels are not essential for intoxication diagnosis^8,9^.

Several tests have been described for the differential diagnosis of RPD. The investigation approach includes electroencephalogram (EEG), brain MRI, ^18^F-FDG PET/CT, cerebrospinal fluid analysis, and AD biomarkers together with the measurement of lithium serum levels^1^. EEG has been described as an important follow-up method. There was an EEG improvement in encephalopathy after drug withdrawal, according to previous reports.

An “Alzheimer-like” pattern of glucose metabolism was found in FDG PET/CT images of the present patient at the initial presentation. This finding was previously reported^2,10^. Interestingly, although there was a marked hypometabolism of the parietal lobes, including the precuneus, and to a lesser extent, of frontal lobes, the temporal lobes and posterior cingulate gyri were spared, differently than previous reports^2,10^. AD was ruled out by screening biomarkers in cerebrospinal fluid, which tested negative. A gradual and complete recovery of parietal metabolism was demonstrated by PET/CT images after lithium suspension. These imaging findings might be related to specific mechanisms of the pathophysiology of acute lithium toxicity in the central nervous system. This also suggests FDG PET/CT as a potential tool for monitoring lithium intoxication.

In this case report, a mild hypometabolism of the frontal lobes persisted after 2 months of lithium suspension. This could be explained by a slower recovery of this region or irreversible lithium neurotoxicity^2^. Furthermore, an additional subclinical neurodegenerative disease cannot be excluded.

In conclusion, lithium intoxication is among the several causes of RPD, and both acute and chronic presentations have been described. Drug withdrawal potentially leads to symptoms regression. Lithium suspension should be considered even in patients with normal serum levels presenting with neurocognitive impairment. ^18^F-FDG PET/CT may identify an “Alzheimer-like” image pattern in acute lithium intoxication and appears to be useful for monitoring these patients, especially in those like this case report, who had negative cerebrospinal fluid Alzheimer’s biomarkers.
